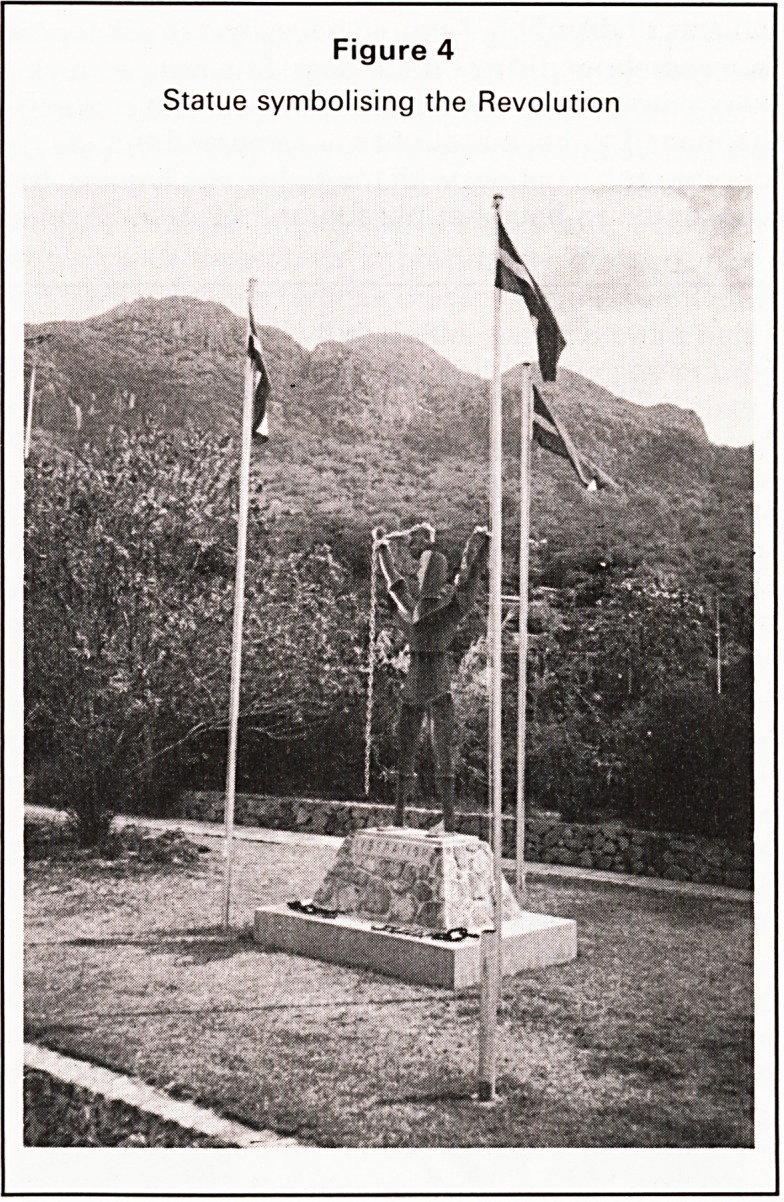# From Our Foreign Correspondent

**Published:** 1984-10

**Authors:** 


### From Our Foreign Correspondent

**Swansong from the Seychelles**

Professor Williamson's letter began 'There may be 101 reasons for your not wanting to visit the Seychelles'. I could not, offhand, think of one. They wanted a locum surgeon. I contacted the Crown Agents as directed and after completing a curriculum vitae and furnishing references was accepted. Eventually, after various delays, I found myself in an aeroplane bound for this little group of islands in the Indian Ocean 1000 miles from anywhere. An airline ticket had been sent me from the Seychelles which on checking had disclosed a non-existent return flight. At Gatwick airport my seat was unconfirmed and I was only allowed on the plane at the last moment. No other instructions had arrived and I wondered as dawn broke and the plane began to descend through intermittent cloud onto what appeared to be forest covered mountains in the sea, what the next move would be. The airfield was built out on land reclaimed from the sea and we landed smoothly. A voice asked us to remain seated until the Health Officer had come aboard, later it said 'Will Dr. Wilson report to the Health Officer'—so I knew I was not forgotten. He turned out to be an engaging young man wearing a Rugby shirt. He led me to the front of the queue. The immigration Officer stamped my card; if I had had my spectacles on I might have noticed that it said I was to do no work, paid or unpaid. The Customs Officer regarded me as an 'interesting case' and carried out a thorough examination. The sporting Health Officer handed me over to a driver and we set off along the coast road. We came in to Victoria and he pointed out to me the entrance to the hospital, to my surprise he drove straight on into the centre of the town where stood a little clock tower, a gleaming silver-painted affair, looking like a huge grandfather clock. He turned out of the town towards the mountains, up a steep winding road and then down the other side. I began to wonder where we were going. Eventually we entered a garden beautiful with flamboyant trees in flower and fragrant from the the lovely waxy blossoms of the franjipani trees. This was the Beau Vallon Hotel. Four dusky damsels were waiting in the foyer with iced lime juice, identically dressed in elegant gowns. Behind the reception desk a charming Creole lady smiled a welcome. I explained who I was and that I had just been brought here and hoped she had heard about me. 'Oh don't worry' she said, 'Go and have some breakfast and I'll ring the Health Department.' After breakfast she said 'They'll come for you when they want you, relax and enjoy yourself—yes—do go on the beach, we'll fetch you when they call'. This was becoming more and more like the Arabian Nights. This charming ministress should have had a name like Scheherezade, but a notice on the desk said she was Mrs. Watson, Duty Assistant Manageress. I have never seen a beach so beautiful, the sand was pale gold, very fine and soft to the feet, coconut palms fringed it and the forested mountains rose up behind. The only building visible was a white church tower peeping up through the trees. I sank into the sea and then fell asleep on the beach in the shade of a coconut palm. I awoke to find a man standing over me. Was I Dr. Wilson? Soon he was driving me to the hospital. He asked if I had my diplomas with me. I said that since they had been handed to me 44 years ago this was the first time anyone had shown any interest in seeing them and anyway it would take too long to go back for them. Fortunately no one else mentioned them.

The Hospital built in 1924 (Figure 1) is a gracious two storey building, whitewashed with blue painted arches, wide decorative iron balconies and built around three sides of a small garden. It houses the administration, the medical wards and the obstetric department. A new hospital is being built which will eventually replace the old building. Phase 1 of the new hospital has been completed and this houses the surgical wards with gynaecology and the operat-

**Figure 1**

Victoria Hospital ing theatres. Phase 2 is in progress and excavations were on all sides. There are 200 beds.

I soon met the outgoing locum who was British, the Belgian Senior Registrar and the Indian Registrar and S.H.O. I soon learned, to my relief, that the Senior Registrar was not only a charming fellow and generally a capable surgeon, but also experienced in Orthopaedics and happy to take care of that side of things. Also, as time went on, I came to appreciate the sterling qualities of the Registrar. To complete my satisfaction I found a British Consultant Anaesthetist, a native of Bristol and a graduate of Leeds (my Alma Mater), on secondment from Londonderry, and a Senior Registrar in Anaesthetics from Aberdeen. They had also taken one of the brightest nurses from the Theatre staff and trained her to give anaesthetics.

The Architects of the new hospital are the British firm of Watkins Gray and Partners and they have produced the best ward and theatre environment it has ever been my pleasure to work in. The wards are divided into 6-bed sections with open access, there are nurses rooms, doctors rooms, large rooms for dressings that almost amount to minor operating theatres, lounge areas for patients and their relatives where they can watch television, all surrounded by balconies with views of the sea on one hand or of the mountains on the other.

The senior Nursing Staff seemed mostly to have had their training in England and all of those I met were I think native Seychelloises. The nursing care was exemplary and the wards immaculate, I can only say that I would have happily been a patient in the male surgical ward under the care of the Sister whose photograph appears with Mr. Dingwall, of whom more later (Figure 2).

The Health Department had printed its own Pharmacocopoea of drugs kept in stock, and while, of necessity, this could not be a duplicate of MIMS, it seemed to contain nearly everything one could expect to need and a wide variety of antibiotics was available. A physician might have taken a different view, but also it might be said that the British Health Service could save itself a mint of money by being more selective in its prescribing.

The range of conditions on the surgical service was wide. It comprised all the usual conditions seen in the UK, plus a tropical element, plus a variety of conditions which at home would go to special departments. A panel of specialist surgeons, e.g. orthopaedic, plastic, ENT come out once or twice a year for two or three weeks and deals with a waiting list of operations and consultations that have been built up for them. Cases requiring radiotherapy are flown to Nairobi or Mauritius and of course all the specialist centres in the world are nowadays readily available by air.

On my first ward round I noted two cases of

**Figure 2**

Sister and Mr Dingwall

Tropical Paraparesis, a condition I had heard about as Seychelles Paraplegia. One was a longstanding case admitted with an acute abdominal emergency and the other admitted for investigation by myelography. I met another case later in the clinic, sent up with an effusion in the knee. He was brought up in a wheelchair and was unable to climb onto the examination couch. The aetiology is unknown. Alex Mellanby, the consultant physician thinks it is multifactorial and some cases clear up with massive doses of vitamins in the form of Parenterovite. The paralysis may affect arms as well as legs and is seldom complete. It comes on rather quickly in a matter of 2–3 weeks. There are a hundred or more cases on the islands and sometimes one sees them out being pushed around in wheelchairs.

In a community where the simple pleasures are uninhibited one might expect venereal disease and its aftermath to be common and there were four cases of complicated urethral stricture. Violence on the other hand seemed to be rare, I recall only two cases, both children—an infant with a compound depressed fracture of skull produced by a stone aimed at his mother and a six-year-old boy with two loops of intestine hanging out of a knife wound inflicted by an eleven-year-old boy who thought he was being cheeky. Other injuries which come to mind were a 6-year-old boy admitted with 6 inches of a fish's beak embedded in his back. He had been out fishing with his father and, standing up in the boat, was struck by a fish flying through the air. A large variety of carcinomas led to the speculation that there is a high incidence of malignancy. Mahé is a granite island and therefore probably has a high level of ambient radioactivity, which in some areas of the world is associated with a high incidence of certain tumours. No evidence of this association has yet been produced for the Seychelles. The acute abdomen was of course common, not only for the usual reasons, but also because worms, especially ascaris, can produce acute abdominal symptoms and mimic obstruction. It seems that half the population of the islands has ascaris, ova in dried faeces become airborne in dust particles and are ingested. Strongyloides also seems to produce an acute upper abdominal syndrome, perhaps by blocking bile or pancreatic ducts. I also saw acutely painful hepatitis without jaundice due to amaebiasis on the medical side as well as an overwhelming liver failure probably due to leptospirosis.

The staff of the hospital was cosmopolitan and this added spice to relations which were most cordial. The Hospital Administrator, the benign lynchpin of the smooth running of everything and a namesake of the President was Seychellois. The Principal Medical Officer, the Pathologist was a Liberian trained in Belgium. The two (heavily overworked) Obstetricians and Gynaecologists were Jugo-Slavian. Two paediatricians were Italian. The Physician was British as had been the surgeons over many years. The Registrars in Medicine, Surgery and O and G were Indian. Consultants generally are appointed for a period of three years. In the old days they would be provided by the Colonial Medical Service. Nowadays I think there is no preference for any particular nationality though lines of communication with Britain are still strong. The Consultant Radiologist is British, recently retired from the Chair of Radiology in Nairobi, and was another reason why working in the hospital was so pleasant. You could always find him in his department, usually in his office in front of an X-Ray screen typing his reports and smoking his pipe. He was a mine of information on all subjects and a close friend of our late lamented Professor Sir Howard Middlemiss.

There were also small hospitals on the islands of Praslin and La Digue. I visited the hospital on Praslin, on the lawn in front the words 'Praslin Hospital' were neatly cut out in dwarf shrubs. The sister on duty took me round. It was trim and spotless as a hospital should be. There was a male ward, a female ward, a maternity ward and a labour ward. There was only one patient, an old man with a swollen leg, allegedly due to Filariasis who was there because there was nobody to look after him at home. But as sister explained it was Sunday and things were much busier during the week.

One of the great joys of being a doctor is that you meet so many people and can have a real relationship with them. In the Seychelles there is the extra dimension of the racial mix. They come in all colours, shapes and sizes. The basic human ingredients are perhaps 50% African, 25% Europen and 25% Asian. These have been mixed up by marriage for 200 years and so you see a dark skin on a European face or a fair skin and even fair hair on an African face, with an infinite number of variations on the three basic themes. Whatever the physiognomy most people seemed bright, relaxed and friendly. Social distinctions seemed slight and unimportant, everyone seemed well dressed or appropriately dressed, particularly the women whose dresses were elegant and colourful and set off with shade-giving picture hats. The way a mother dresses her child when she brings her up to hospital tells you much about a society. Seychelles mothers scored full marks on this one. Indelibly imprinted on my mind is a beautiful little girl of about 7 with beribboned pigtails and a pretty printed dress. A few days previously her mother had noticed an opacity in her left eye, which was also blind—no one knew for how long. She had come via the Medical side from the Eye Clinic (run by an ophthalmic nurse, there being no ophthalmologist on the island) with a diagnosis of ?Retinoblastoma and a suggestion that she ought to have an enucleation. None of us had any experience of this highly malignant condition, but the testbook description seemed to fit the case. I shrank from performing such an act. Fortunately we were able to fly her to Nairobi where a diagnosis of congenital cataract was made.

I must also mention two other unforgettable people. The first a patient in the male ward. He was there when I arrived and there when I left a month later and I saw him nearly every day. Two years before he had had an abdomino-perineal resection for carcinoma of the rectum. Now he has extensive recurrence and the groins are solid on both sides, the scrotum and both legs are oedematous. He has continuous pain due to nerve involvement but is kept comfortable with morphia. Sometimes he would be asleep, sometimes asking for his next injection, but with liberal analgesia in anticipation of pain he would come alive. One day he showed me a photograph. It was of the the Duke of Edinburgh inspecting a Guard of Honour of Seychelles ex-servicemen, accompanying him, with a chest full of medals was Corporal Dingwall. He served in North Africa and acted as coxswain in some kind of amphibious outfit. He was mentioned in dispatches for gallantry in action. He let me photograph him with his photograph and it gave me an excuse to get a photograph of sister as well. I think it is one of the nicest photographs I have ever taken (Figure 2). My last unforgettable character, though I could mention so many others, must be Mother Theresa. One morning, while between cases in the theatre, we heard that Mother Theresa was in the hospital, in fact she was just going in to the Surgical Wards. I went along and joined the party with her. She appeared to be in her seventies and was rather bent, but she was quick and alert. She spoke in English to many of the patients and gave them medallions as souvenirs and objects of devotion. I should have liked to ask for one but somehow did not quite dare. She noticed one or two empty beds and said how lucky we were. I happened to have brought my camera in that day to photograph a patient with multiple huge keloids. She consented to have her photograph (Figure 3) taken

**Figure 3**

Mother Theresa visits the Surgical Wards and we formed a group round her—then she swept on. My fleeting impression was of a woman, who, for her age, was still extremely active and yet remained calm and serene. In repose her face was rather sad, but when she spoke it lit up with kindness and interest. I was surprised to learn that she was Albanian but had gone to India when she was a child and had forgotten her native language.

Mahé is the largest of the islands, of which there are 115, about 1000 miles away from the nearest worthwhile landfall which is Mombasa. Victoria, the capital is on Mahé which has a population of about 60,000. Praslin has about 6000, La Digue 2000 with another thousand or so spread over the remainder (if you discount the North Korean troops rumoured to be stationed on the outer islands.) The main islands are granitic outcrops rising to nearly 3000 feet on Mahé and heavily wooded. Praslin is the only naturally occurring site of the Coco-de-mer which bears a double coconut, the largest nut in the world. For centuries these nuts had been found washed up on the shores of the Indian Ocean and, as they had never been seen growing on any tree it was thought they must grow in the sea—hence the name. There is a forest of these huge trees on Praslin, the double coconuts resemble the female genitalia and the large male catkin is remarkably like the phallus. When General Gordon came to the Seychelles he thought it was the site of the garden of Eden and that the Coco-de-mer was the Tree of Life. The nuts, of course have been esteemed as aphrodisiacs.

The sea around the islands is full of fish, easily caught. Bananas, melons, paw-paws, bread fruit, tomatoes, pineapples etc. grow in abundance, there are coconuts galore and so there is toddy. A simple subsistence is possible without much back-breaking toil. We watched traditional fishing from Beau Vallon beach. The pirogue is a simple, long wooden rowing boat. Two of these, with four oarsmen each and a steersman standing up in the stern with another oar go out from the beach for a few hundred yards. They then row apart from each other parallel to the shore each pulling one end of a long net. When it is straightened out they turn and make for the same point of the shore, bringing the net round in a great circle. When they have reached the shore they leap out and start pulling the net in. Always they had some fish, sometimes the catch was enormous with a huge variety of fish—large tuna, mackerel, eels, octopus. Most of them I did not recognise, including a funny little sword fish about 3 feet long, half of its length being beak, this was probably the species that embedded itself in the little boy as described above. Most of the time the fishermen were sitting round smoking cigarettes and waiting for favourable omens. At the other end of the scale tourists were taken out in large speedboats with two huge outboard motors carrying rods baited with large balls of nylon yarn. Marlin and sailfish would pursue these balls and dig their long beaks into them. The helmsman would then accelerate, this would tighten the nylon round the fish's beak and then it was simply a matter of playing the fish until it was exhausted and then reeling it in. These marvellous fish, 6 feet or more in length, would be brought in and hoisted over the branch of a tree so that the proud victors could be photographed standing beside them. They were delicious eating, especially when smoked. Trawlers of various sizes plied their trade from Victoria harbour. The counters in the market were loaded with a profusion of fish of astonishing variety. On the trees above egrets perched, ready to swoop down at an auspicious moment. Not surprisingly, every few days, a patient would come to the hospital convinced he had a fishbone stuck in his throat. If present, an X-Ray would usually show it, but more often than not there was nothing to see and the patient would be asked to come back next day. Usually the pain had gone, presumably the bone would puncture the pharynx in the act of swallowing and then pass on leaving a painful wound.

The islands were ininhabited until the French planted the first colonies in 1760. In 1814, at the Treaty of Paris after the defeat of Napoleon, they were ceded to Britain. The French Governor continued to administer them until he died in 1827. Seychelles began to emerge from the colonial era in 1964 when the franchise for the Constituent Assembly was enlarged and political parties began to emerge. The Seychelles People's United party was formed by Albert René with a policy of non-aligned socialism and a one party state like Tanzania. In response James Mancham formed the Seychelles Democratic Party supporting the continuation of the capitalist free enterprise system and maintaining close links with Britain. His aim was to encourage tourism and make Seychelles a kind of Monaco in the Indian Ocean. Mancham's party won three elections and when the country was given internal self government in 1975 he became it's first Prime Minister in a coalition with René as Minister of Works and Development. The coalition held and in the following year they achieved full independence. Mancham became President and René, Prime Minister. A year later Mancham flew off to attend his first Commonwealth Conference as Head of State. René kissed him goodbye at the Airport. He wasted no time and, with the aid of Tanzanian troops, got on with his Revolution. Perhaps for the first time in the history of the islands power was seized with the shedding of blood, a very little blood and no doubt much regretted, but accidents will happen even with the best conducted revolutions. The Seychelles now has a one party socialist state dedicated to the promotion of social equality, welfare and education. René has survived two counter coups. Tanzanian troops remain, but in the background. To create a corps of men and women devoted to his ideals René has established a National Youth Service. All boys and girls between 16 and 18 are obliged to spend these years in camps, wear uniform, submit to rigorous discipline and indoctrination. During this period they are allowed to spend only a few weeks with their families. Does one equate this with the Hitler Youth, with the Boy Scouts and Girl Guides or with the British Public School system? At the very recent Presidential Election the voters had the chance of voting 'Yes' or 'No' for René. There was no alternative candidate and the ballot papers had serial numbers. He was returned with an overwhelming majority.

What does one make of all this? I avoided talking politics and got the impression that most other people do too. Certainly one feels that the people are getting an excellent deal on health care and education. René is spoken of as being high minded and unselfishly dedicated to the people's welfare. Mancham as being pleasure loving and corruptable.

**Figure 4**

Statue symbolising the Revolution

It is sad however to see the pendulum swing away from democracy. To celebrate the Revolution a particularly grim looking piece of steel-work was erected (Figure 4) showing a sort of robot man holding aloft chains. Whether he was taking them off or putting them on history has yet to show. Actually the whole atmosphere of the islands is so pleasant, the people so friendly, nature so benign, the possibility of starvation so remote that, at any rate during René's lifetime one can hope that the islands will enjoy a regime of enlightened paternalism.

M. G. WILSON

## Figures and Tables

**Figure 1 f1:**
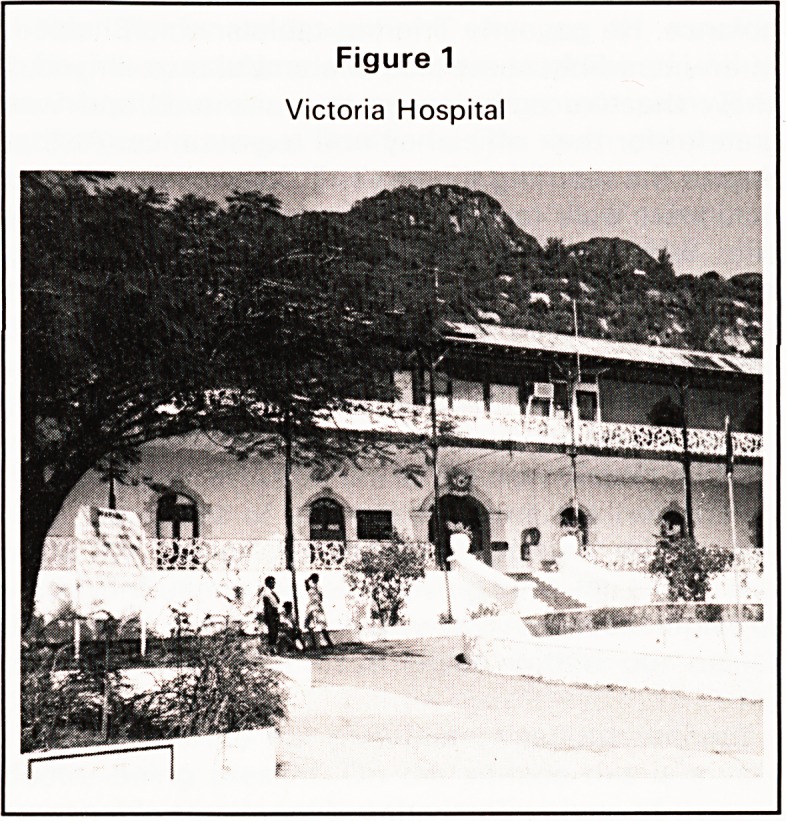


**Figure 2 f2:**
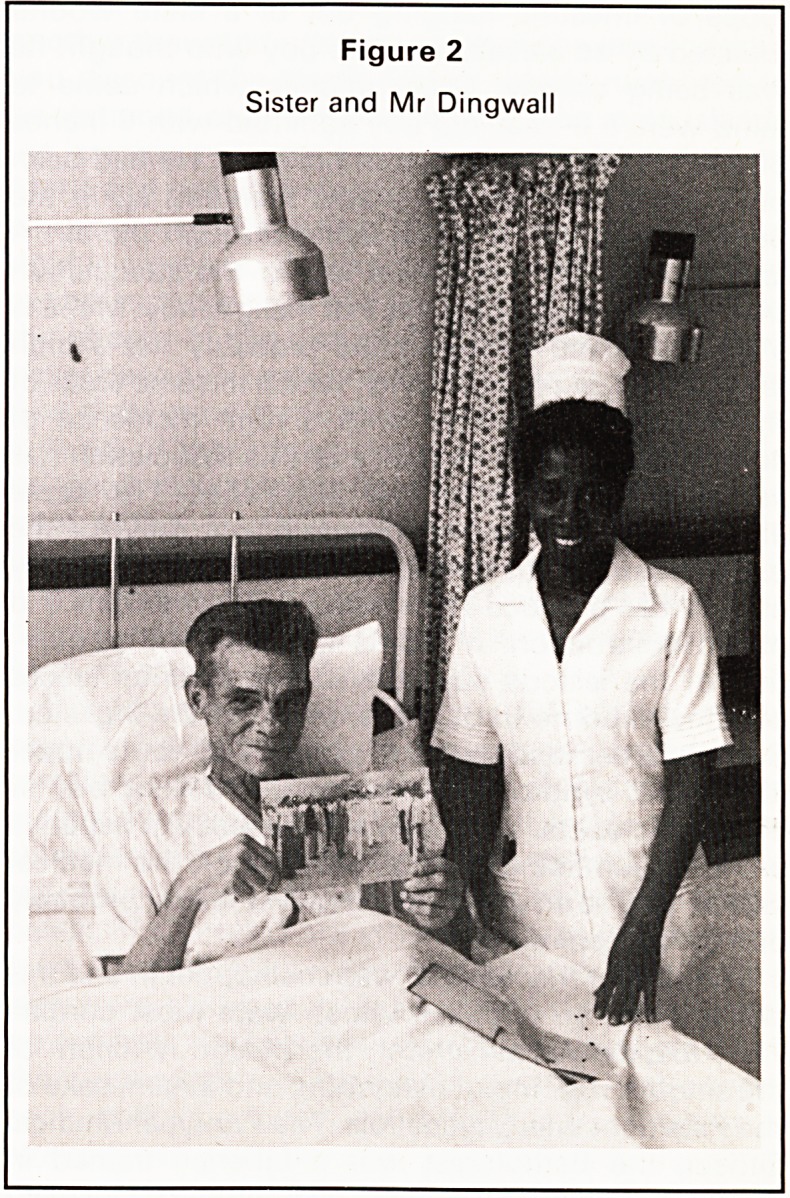


**Figure 3 f3:**
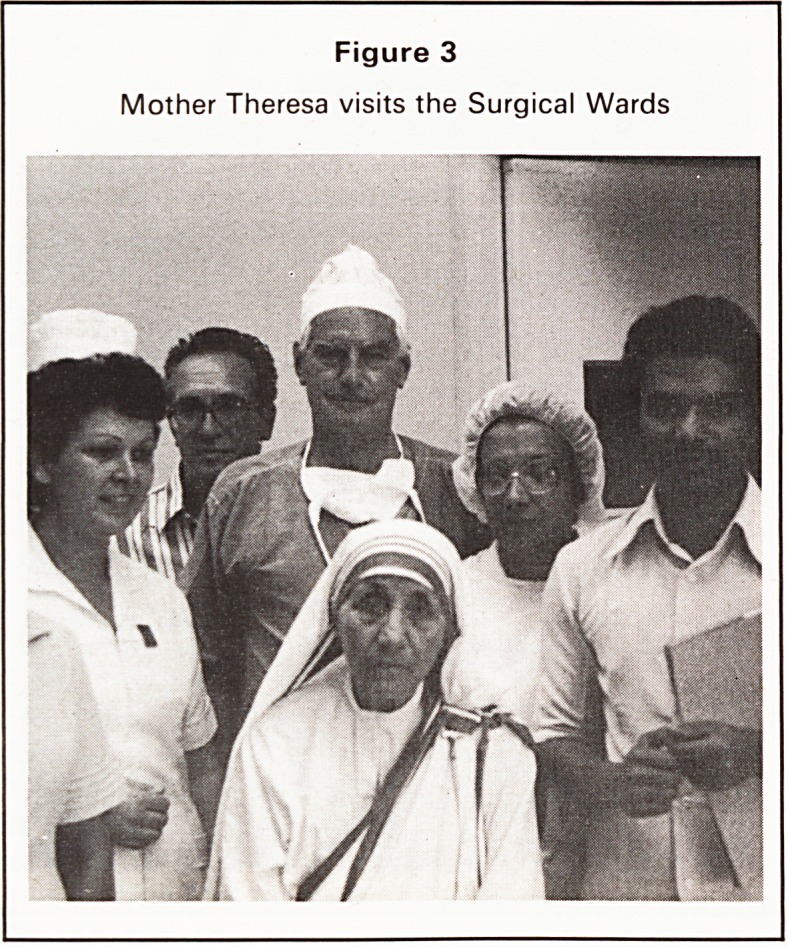


**Figure 4 f4:**